# A numerical investigation of the behavior of a tunnel with adaptive anti-dislocation measures subjected to the action of fault dislocation

**DOI:** 10.1038/s41598-024-51445-5

**Published:** 2024-01-12

**Authors:** Fajiang Bi, Xiao He, Yanjie Zhang, Zhen Cui, Xiancheng Mei, Jianhe Li

**Affiliations:** 1 Construction Administration Bureau of Central Yunnan Water Diversion Project, Kunming, 650205 China; 2 Central Yunnan Water Diversion Project Co., Ltd, Kunming, 650000 China; 3 State Key Laboratory of Geomechanics and Geotechnical Engineering, Institute of Rock and Soil Mechanics, Chinese Academy of Sciences, Wuhan, 430071 China; 4 Key Laboratory of Changjiang Regulation and Protection of Ministry of Water Resources, Changjiang Engineering Group, Wuhan, 430010 Hubei China

**Keywords:** Engineering, Civil engineering

## Abstract

In the context of long-distance cross-basin water transfer projects, the water conveyance tunnel serves as a pivotal component in mitigating regional disparities between economic development and water resources allocation. However, in high seismic-intensity areas of southwest China, geological complexities and densely distributed active faults present formidable challenges. Consequently, the construction of water conveyance tunnels necessitates traversing one or more active fault zones. This study examines the impact of an adaptive tunnel structure in the presence of fault dislocation, focusing on the Xianglushan Tunnel, a constituent of the Central Yunnan Water Diversion Project. Taking the Longpan-Qiaohou Fault F10-1 as a case study, we assess the influence of active faults on the anti-dislocation adaptive structure of the Xianglushan Tunnel, considering factors such as displacement, relative deformation, maximum principal stresses, and longitudinal equivalent internal force in critical tunnel sections. Numerical calculations validate the efficacy of this adaptive structure in reducing induced internal forces and deformations of the tunnel lining. The results show that, under the influence of strike-slip dominated fault movement, one side of the tunnel exhibits tensile stress, with a magnitude of approximately 5 MPa. The maximum normal and tangential deformation of the hinge joint is concentrated in the central section of the fault zone. The incorporation of an articulated adaptive design significantly enhances the stress state of lining under dislocation condition. These research results directly inform the engineering design and construction of water conveyance tunnels traversing active fault regions, providing valuable guidance for related tunnel construction endeavors.

## Introduction

With the gradual improvement of Chinese economic level, substantial progress has been achieved in mitigating the regional disparity between economic development patterns and the allocation of water resources. The water conveyance tunnel, a central component of long-distance cross-basin water transfer projects, assumes a pivotal role in addressing this regional incongruity. Nevertheless, the intricate geological composition and the dense prevalence of active faults in the high seismic-intensity area of southwest China pose significant challenges. This is particularly evident in the context of the Central Yunnan Water Diversion Project, which seeks to fundamentally solve water scarcity issues in central Yunnan, and consequently necessitates traversing multiple active fault zones. These active fault zones pose a serious threat to the safe operation of the tunnel project, thereby underscoring the urgent and imperative need for a comprehensive investigation into the anti-dislocation design of tunnels crossing such active fault zones. The most famous tunnel impairment due to active fault creep is exemplified by the Claremont Water Tunnel, wherein movement along the Hayward fault led to a 13-inch dislocation over a period exceeding 80 years of service. Consequently, the bypass of the tunnel segment passing through active faults was reconstructed in 2006. This extensive endeavor, involving a 500 m bypass reconstruction, extended over a duration of three years and incurred a total cost of $34 million.

Active fault zones are potential seismogenic zones that are susceptible to dislocations due to geotectonic force, thereby giving rise to seismic events^1^. Meanwhile, stick–slip active faults exhibit sudden and rapid energy release, resulting in instantaneous dislocation, which can lead to severe damage to engineering structures. Recognizing the potential movement of bedrock faults as a significant threat to existing tunnels located in seismically active areas, Cai^2^ conducted centrifugal model tests to investigate the deformation mechanism of existing tunnels experiencing normal fault dislocation. The study also addressed the effects of different tunnel boundary conditions and lengths on the response of lining structures under the action of normal fault dislocation. Burridge et al.^3^ conducted a series of centrifuge shaking table tests on a limited-length tunnel model, allowing for a quantitative analysis of the bending and stress response induced by fault dislocation during passage through a fault zone. Liu et al.^4–6^ simulated the stress-deformation response characteristics of tunnels orthogonal to a normal fault with dip angle of 45°, 60°, and 75° respectively, under the action of stick–slip dislocation, employing an indoor flask test with a similitude ratio of 1:50. As for the case of Sabzkouh Tunnel crossing the Sulaghan Fault in Iran, Ghadimi et al.^7^ used numerical methods to study the influence of tunnel location, lining thickness, intersection angle between the fault and the tunnel, mechanical properties of the surrounding soil and the fault dip angle on tunnel stability under the action of reverse fault dislocation. Jiao^8^ used finite difference software to establish a corresponding numerical model for an actual tunnel project, shedding light on the effects of earthquake-induced inverse fault dislocation on tunnels crossing the active faults orthogonally and revealing its underlying influence mechanism. Jeon et al.^9^ discussed the influence of weak contact surface between the tunnel lining and the surrounding fault rock on the lining structure through numerical analysis. This comprehensive analysis attests to the substantial progress achieved in elucidating the damage mechanisms and evolution law of tunnels passing through active fault zones.

However, studies on adaptive anti-dislocation design of tunnels crossing active fault zones are relatively scarce, mainly focusing on engineering experience and conceptual design, such as articulated design^10^, over-excavation design^11,12^, isolation efficiency design^13^ etc., Among them, the most widely applied “articulated design” is aimed to design the structure into one with the same characteristics of a chain hinge, thus forcing the structure to move in a hinged way under the action of fault dislocation, Then the structure can absorb and dissipate the deformation through the sliding hinged segment that can slide and twist along the tunnel axis, so that the damage will be concentrated in the connection part, thus avoiding overall damage to the structure^14,15^. The Claremont Tunnel in San Francisco crossing the Hayward Fault once faced the threat of fault dislocation^16^. Its lining is hinged, with a 0.3 m-wide shear joint reserved for every 1.5 m long lining segment, in assuming that the lining can absorb the deformation caused by the fault shear under the action of fault dislocation, thus avoiding the overall damage to the tunnel. The Koohrang-III water conveyance tunnel in central Iran crosses at least four fault zones, the largest of which is the Zarab Fault. To reduce the strength and stiffness of connecting materials, absorb the deformation caused by fault dislocation and protect the lining structure, measures are taken to set steel lining in the inner layer and set plastic concrete connecting sections between lining segments^17^. From a theoretical perspective, it is possible to select appropriate design parameters in a hinge design based on the potential fault movement, and achieve targeted active fortification; from the perspective of construction, in tunnel construction, only the construction steps of articulated segments are added, which is convenient for implementation; in addition, from the perspective of effectiveness, in the case of large range of fault dislocation, the damage to the tunnel can be localized as the strength and stiffness of the flexible connection materials at the articulated segments are low, thus avoiding overall damage to the tunnel and reducing the cost of maintenance and repair^18^.

To date, a lack of standardized specifications and norms governing the design of anti-dislocation measures for tunnels passing through active fault zones has necessitated a rigorous evaluation process in anti-dislocation design. This study focuses on examining the influence of fault dislocation on the tunnel adaptive structure, employing the case of the Xianglushan Tunnel, which crossing active fault zones within the Central Yunnan Water Diversion Project. Taking the Longpan-Qiaohou Fault F10-1 as a typical case, this paper evaluated the influence of the active fault on the anti-dislocation adaptive structure of the Xianglushan Tunnel from the aspects of displacement, relative deformation, maximum principal stress, longitudinal equivalent internal force and other factors of the key tunnel parts. The research results can be directly applied to the engineering design and construction of water conveyance tunnels crossing active faults, and furnish a substantive foundation for the development of related tunnels.

## Background project

The Central Yunnan Water Diversion Project constitutes a pivotal strategic infrastructure initiative executed in China, with the primary objective of optimizing the water distribution in Yunnan province and alleviating water scarcity in the Central region. Upon completion, this project is anticipated to provide a sustained mitigation of water scarcity in Central Yunnan, improve the ecological and hydrological conditions of rivers and plateau lakes in the recipient area, and play an important role in promoting the coordinated and sustainable economic and social development of Yunnan Province. It is estimated that by the design level year of 2040, the annual average water diversion volume of this project in Central Yunnan is expected to reach 3.403 billion cubic meters (as measured at the head works), including 2.231 billion cubic meters for urban domestic and industrial usage, 500 million cubic meters for agricultural irrigation and 672 million cubic meters for lake water replenishment.

**Figure 1:**
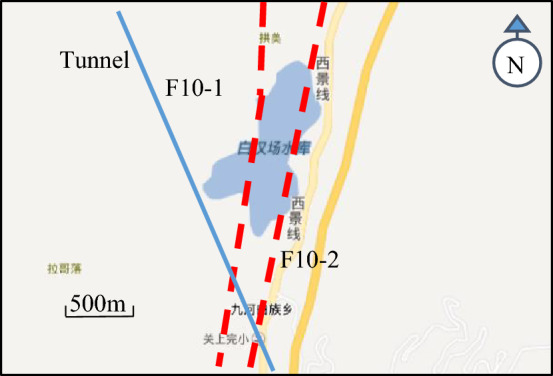
Distribution of Longpan-Qiaohou fault and profile of water diversion line axis.

Xianglushan Tunnel constitutes the linchpin of this extensive cross-basin water diversion (transfer) project, and stands as a representative project of long-distance water conveyance tunnel in China. Spanning a length of 63.426 km, with a maximum burial depth of 1450 m. The total length of the tunnel segments with a buried depth greater than 1000 m is 21.427 km, accounting for 34.23% of the total length of the tunnel. Additionally, the total length of the tunnel segments with a buried depth greater than 600 m is 42.175 km, accounting for 67.38% of the total length of the tunnel. The geological terrain in the vicinity of the Xianglushan Tunnel is notably intricate, with several Holocene regional active faults tracing the route, including the Longpan-Qiaohou Fault (F10) (as shown in Fig. 1), Lijiang-Jianchuan Fault (F11) and Heqing-Ergyuan Fault (F12). For the 100 year displacement fortification due to Holocene active faults, the horizontal vector value is 1.50–2.20 m and the vertical vector value is 0.26–0.34 m. The safety construction and operation of the tunnel are gravely imperiled by these active faults. Therefore, undertaking studies elucidating the mechanisms of dislocation-induced damage and anti-dislocation measures in water conveyance tunnels passing through active fault zones holds profound scientific and engineering import.

## Modeling and parameters

**Figure 2:**
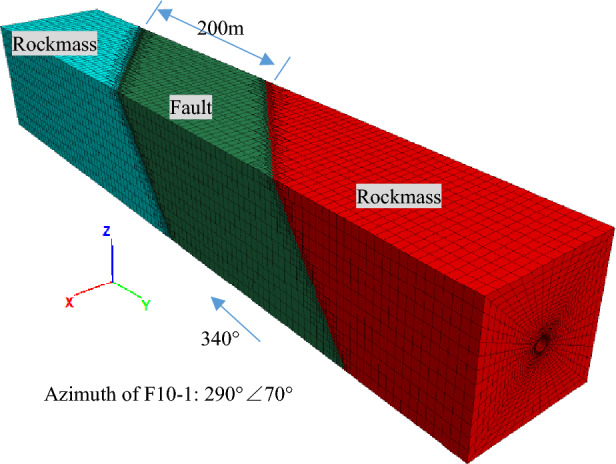
Three-dimensional analysis model of Longpan-Qiaohou Fault F10-1.

The Longpan-Qiaohou Fault F10-1 was used as a typical representative fault to analyze the anti-dislocation design of tunnels. The local 3D analysis model containing the tunnel crossing the Longpan-Qiaohou Fault F10-1 was built in FLAC3D, as shown in Fig. 2. The numerical model, addressing a solitary main fault zone, was established with the tunnel’s axial direction (340°) as the Y axis and the vertical direction as the Z axis. The numerical model featured a spatial extension of 100 m in both positive and negative x-directions, centered on the tunnel’s midpoint, 300 m along the y-axis (aligned with the tunnel’s axial direction), centered on the main fault zone, and 100 m in the z-direction, centered on the tunnel’s midpoint. In the context of the tunnel segment crossing F10-1, a burial depth of 400 m was considered, and the corresponding vertical stress was imposed at the top according to the weight of the upper rock mass in the numerical model. The radius of net tunnel flow section is 4.6 m, and the initial support shotcrete and secondary lining of the tunnel are collectively considered as a 1.05 m-thick concrete lining. This paper mainly focused on the creep-slip fault, addressing issues from a static force perspective, so rendering the load associated with movable displacement as a static load acting on the boundary of the moving wall in the model.

**Figure 3:**
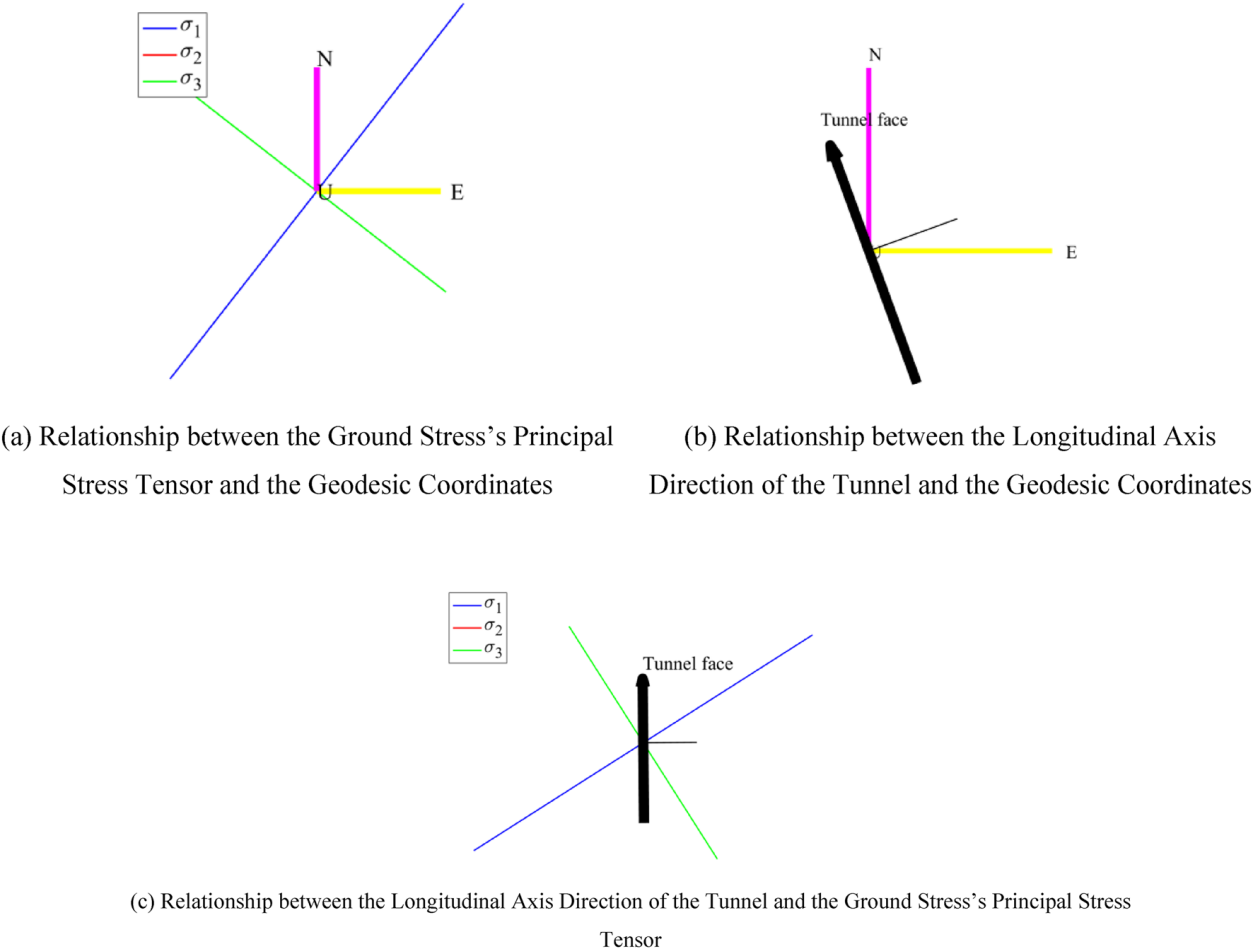
Relationship between the regional ground stress field direction and the tunnel axis.

According to previous researches on the inversion of ground stress field in engineering area^19^, the inverted ground stress field detailed in Table 1 will be employed as a foundational framework in the structural adaptability assessment study for the Longpan-Qiaohou fault area. The relationship between the direction of the ground stress field and the tunnel is shown in Table 2 and Fig. 3. It can be seen that, the intersection angle between the maximum principal stress of the ground stress field and the longitudinal axis of the tunnel is substantial, exerting a profound impact on the tunnel stability.

**Table 1:** In situ stress fields used in current study.

Burial depth/m	Maximum principal stress/MPa	Maximum principal stress azimuth/°	Medium principal stress/MPa	Minimum principal stress/MPa
400	16	40	13	11

**Table 2:** The relationship between the direction of ground stress field and the direction of tunnel axis.

Azimuth of tunnel axis/°	Maximum principal stress azimuth/°	Angle between the maximum principal stress and the tunnel axis /°	Component of ground stress in the tunnel cross section/MPa*						
			Burial depth/m	${\sigma }_{xx}$	${\sigma }_{yy}$	${\sigma }_{zz}$	${\tau }_{xx}$	${\tau }_{yy}$	${\tau }_{zz}$
340	40	60	400	14.75	12.25	13.00	2.17	0.00	0.00

The coordinate convention on the cross-section of the tunnel is: $x$ to the right, $y$ into the plane, and $z$ to the vertical.

For the mechanical parameters of the rock mass in the Longpan-Qiaohou Fault (F10-1) area, the mechanical parameters in Table 3, which estimated by geologists based on engineering experience and laboratory tests, were used in the analysis.

**Table 3:** Mechanical parameters of rock masses used in current study.

Position	Rock type	Mechanical parameters				
		Elasticity modulus/GPa	Poisson’s ratio	Friction coefficient	Cohesion/MPa	Tensile strength/MPa
Hanging wall	IV	3.00	0.30	0.65	0.55	0.25
Fault zone	V	0.80	0.34	0.50	0.40	0.15
Foot wall	IV ~ V	1.50	0.33	0.55	0.50	0.20

## Consideration of the adaptive measures

The articulated design of the lining involves critical design parameters include lining segment length, connecting section length, fortification length, and connecting section’s filling materials. Of these, the most important design parameters are the lining segment length and the connecting section length. For these two design parameters, there is no mature and common design method, but scholars have proposed estimation methods based on research pertaining to as-built tunnel traversing active faults. In this section, two such estimation methods will be used to estimate the lining segment length, connecting section length and other pertinent design parameters relevant to the Xianglushan Tunnel.

### Shahidi & Vafaeian’s estimation method

**Figure 4:**
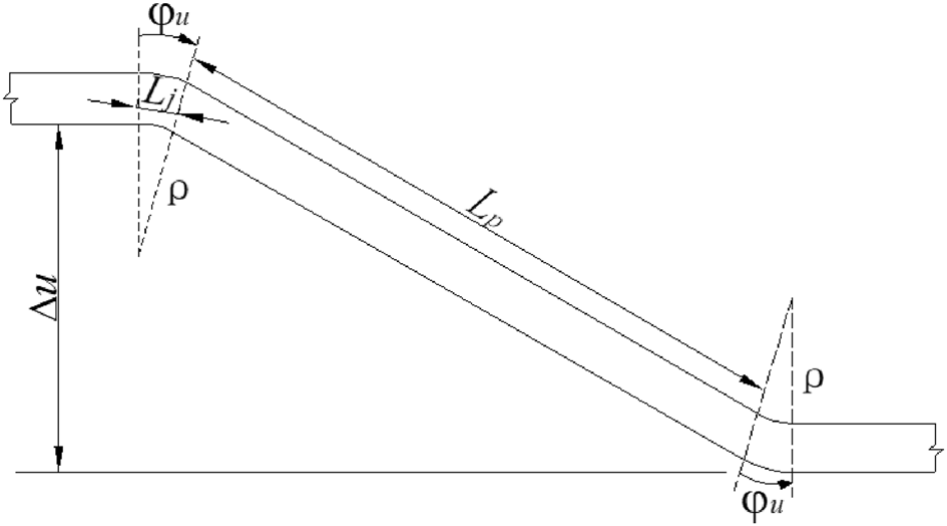
Schematic diagram of the axis deformation of articulated lining^17^.

Shahidi & Vafaeian proposed an estimation method for the articulated design in the anti-dislocation study of the Koohrang-III Tunnel in 2005^17^. The fundamental concept is as follows: Following a fault dislocation event, the tunnel’s lining structure undergoes an “S” shaped bending in the vertical plane, with axial deformation illustrated in the Fig. 4. Here, $\Delta u$ denotes the vertical displacement resulting from fault dislocation, ${L}_{p}$ signifies the segment length, ${L}_{j}$, represents the width of the flexible connection, and ${\varphi }_{u}$ denotes the ultimate bending curvature of lining under the action of surrounding rock conditions during fault dislocation.

Then, the relationship between the fault dislocation range and the lining segment length and the flexible connection width can be expressed as the following formula.

1 $${L}_{j}=\frac{\Delta u}{{\varphi }_{u}\cdot {L}_{p}}$$

where, $\Delta u$ is the vertical fault dislocation range; ${L}_{p}$ is the lining segment length; ${L}_{j}$ is the flexible connection width; ${\varphi }_{u}$ is the ultimate bending curvature of the lining segment.

This present estimation formula neglects the requirement for establishing multiple articulated segments within a broad fault zone, a deficiency that will be rectified in this investigation. When the consideration of multiple hinged segments is warranted, it remains imperative to ensure the fulfillment of the following equation:

2 $$\frac{\Delta {u}_{{\text{F}}}}{\Delta u}\le \frac{{L}_{F}}{{L}_{j}+{L}_{p}}$$

Here, $\Delta u$ is the tolerable dislocation of each articulated segment; $\Delta {u}_{{\text{F}}}$ is the total dislocation of the fault zone; ${L}_{F}$ is the width of the fault zone.

As for ${\varphi }_{u}$, Shahidi & Vafaeian did not give an estimation method, but Jalali^15^ proposed a simplified estimation method as follows.

According to the above formula, and given a tunnel fault width of 200 m, a fortified dislocation range of 40 cm, and a tunnel diameter of 10 m, it can be deduced that for a fortified segment length of 6 m, the requisite minimum width for the articulated segment is approximately 10 cm.

### Jalali’s estimation method

**Figure 5:**
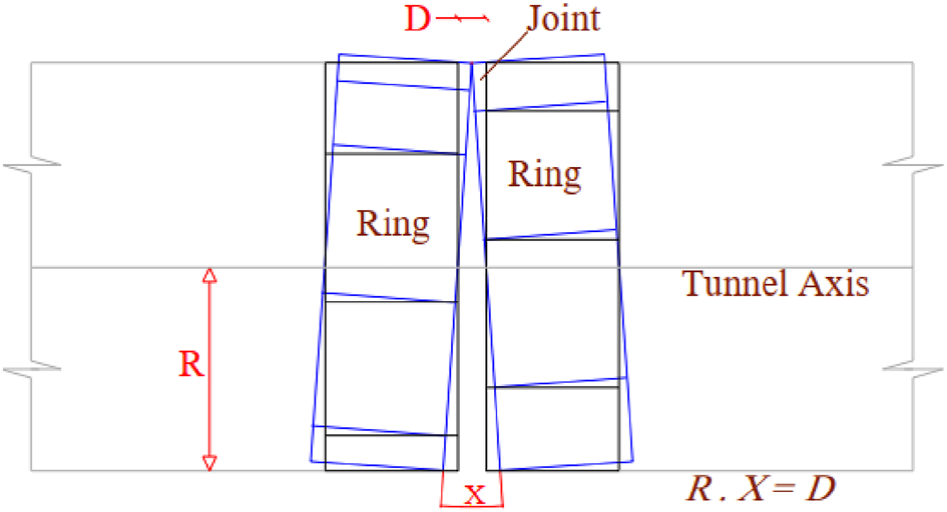
Calculation method proposed by Jalali for the ultimate bending curvature of the articulated segment.

Jalali^15^ proposed another estimation method for the articulated design considering displacement mode in 2018 for the Karaj Tunnel. The fundamental concept is as follows:

The tunnel structure traversing the fault zone was conceptualized as a clamped beam fixed at both ends with uneven settlement of support, as shown in Fig. 5. This beam had infinite stiffness or large finite stiffness, with no or limited bending capacity, and all or most of the corners were located at the “articulated position” arranged at specific spacing along the beam.

For the clamped beam fixed at both ends, when there is a vertical displacement at one end, the deflection curve equation of the beam is:

3 $$\frac{\Delta {u}_{{\text{F}}}}{\Delta u}\le \frac{{L}_{F}}{{L}_{j}+{L}_{p}}$$

where, $a$ is the vertical displacement, $l$ is the length of the beam, and $x$ is the position of a point on the beam in the coordinate system.

**Figure 6:**
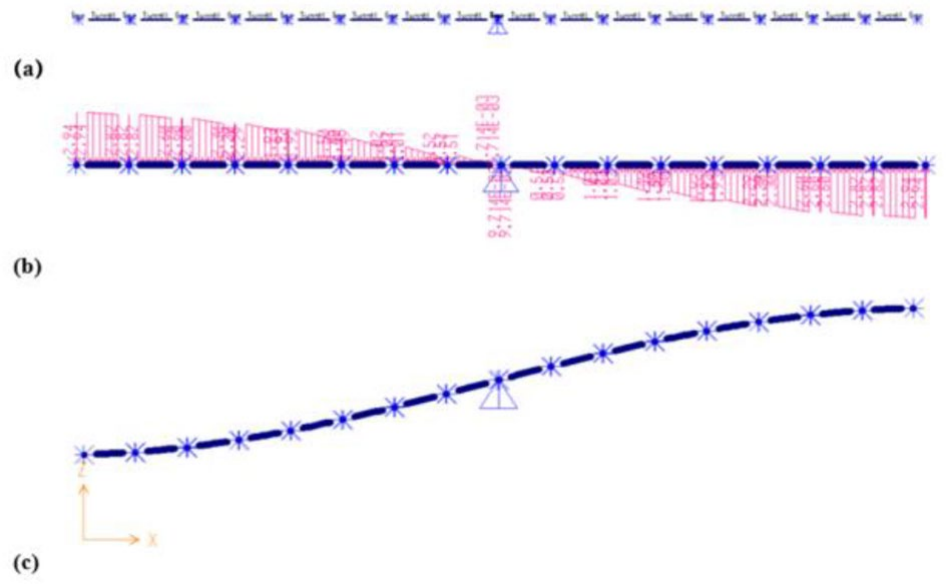
Calculation model proposed by Jalali^15^.

The deflection curve of the forced beam is similar to the theoretical deflection curve of a clamped beam fixed at both ends with uneven settlement of the supports, and the corner at the “articulated position” on the beam is the corner that needs to be satisfied at the articulated segment with respect to the current hinge spacing. The estimated width of the dislocation joint at this articulated position can then be derived from Fig. 6.

By employing the aforementioned method, given a tunnel fault width of 200 m, a fortified dislocation range of 40 cm and a tunnel diameter of 10 m, it can be determined that for a fortified segment length of 6 m, the requisite minimum width of the articulated segment is approximately 4.2 cm. Conversely, for a fortified segment length of 10 m, the minimum required width for the articulated segment is approximately 7.7 cm.

In light of these two estimation formulas, under the same conditions of a 200 m tunnel fault width, a 40 cm fortified dislocation range, and a 10 m tunnel diameter, when the length of the fortified segment is fixed at 6 m, the minimum width of the articulated segment is projected to fall within the range of 4.2 to 10 cm.

## Anti-dislocation design check of tunnels passing through active faults

The anti-dislocation measures considered are as follows: a segment length of 6 m; a hinged joint width of 5 cm; a fortified length extending 1.5 times the fault width, signifying that for a fault zone measuring 200 m in width, it extends 50 m into both the hanging wall and foot wall directions. The hinged joint is filled with plastic concrete materials with an elastic modulus of 500 MPa. In the calculation, the “S” displacement mode with the applied active fault displacement proposed in this study was used^20^, in which the maximum fortification value is considered to be 60 cm horizontally and 12 cm vertically. The reason why the value of 60 cm was used is to more conservatively check whether the design parameters can meet the requirements.

### Overall deformation and damage trend of tunnel

**Figure 7:**
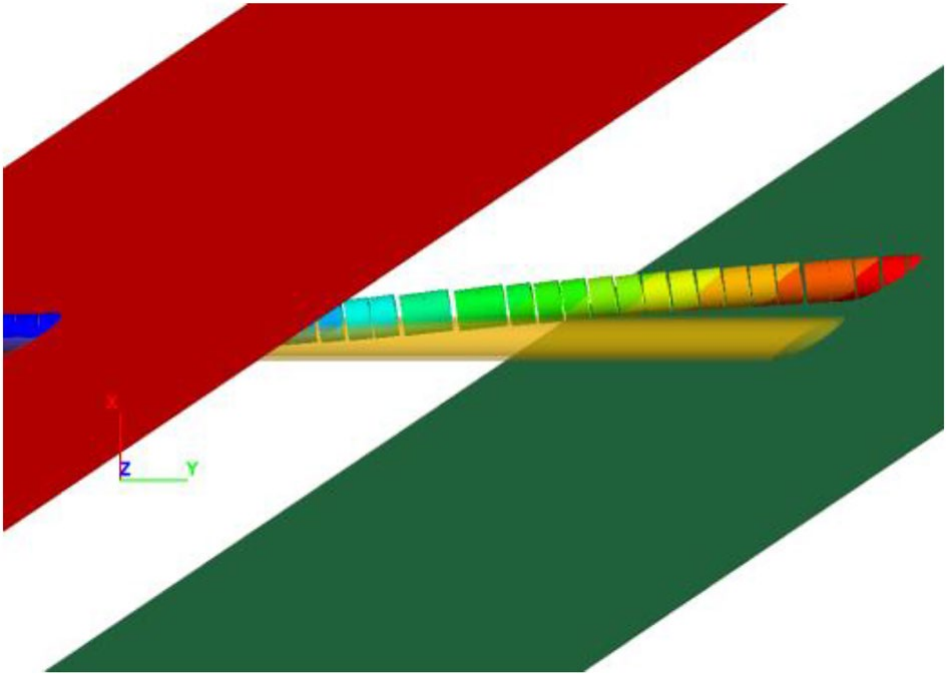
Deformation pattern of the tunnel under 60 cm fortified dislocation.

Figure 7 shows the deformation pattern of the tunnel with a fortified dislocation value of 60 cm. Upon amplification by a factor of 50, it can be clearly seen that the articulated design of the tunnel has played a significant role—all hinged joints exhibit rotational and stretched state, indicative of their effective functioning.

**Figure 8:**
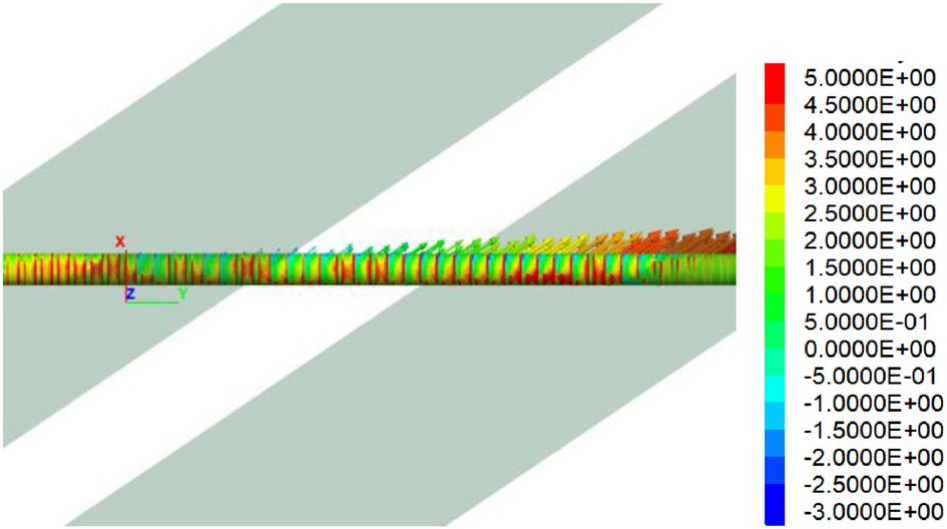
Longitudinal stress of the tunnel under 60 cm fortified dislocation.

**Figure 9:**
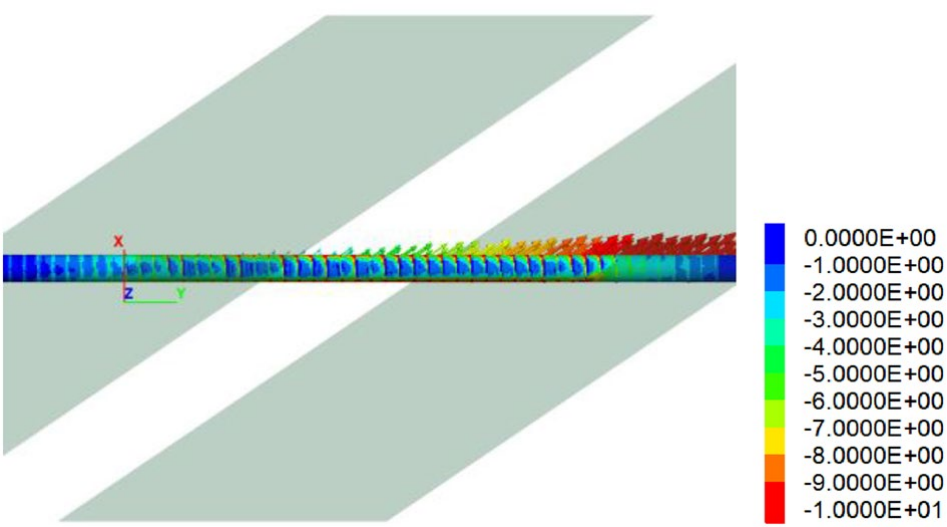
Maximum principal stress of the tunnel under 60 cm fortified dislocation (tension + compression−).

**Figure 10:**
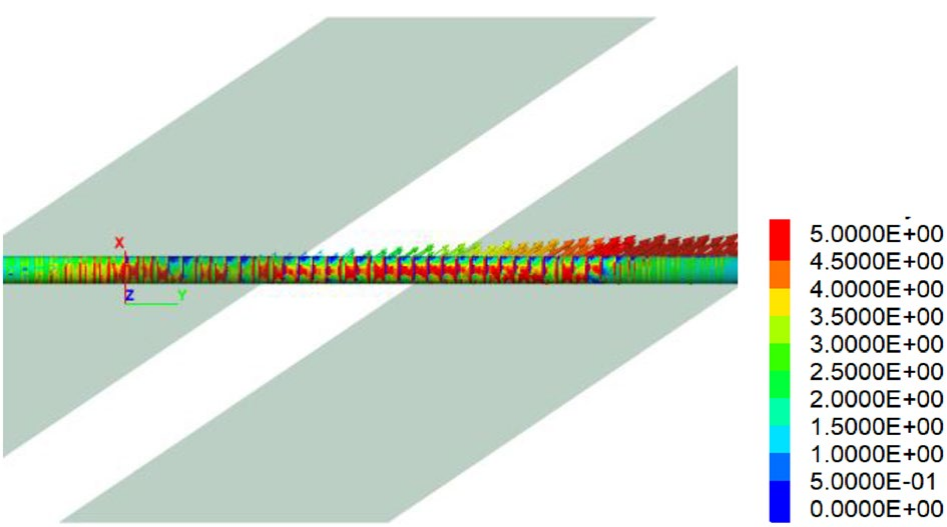
Minimum principal stresses of the tunnel under 60 cm fortified dislocation. (tension + compression−).

Figure 8 is the longitudinal stress cloud chart of the tunnel, while Figs. 9 and 10 is the maximum principal stress cloud chart and the minimum principal stress cloud chart of the tunnel lining, respectively. Notably, one side wall of the tunnel experiences tension primarily due to the predominant strike-slip fault movements, with a tensile stress value of approximately 5 MPa.

**Figure 11:**
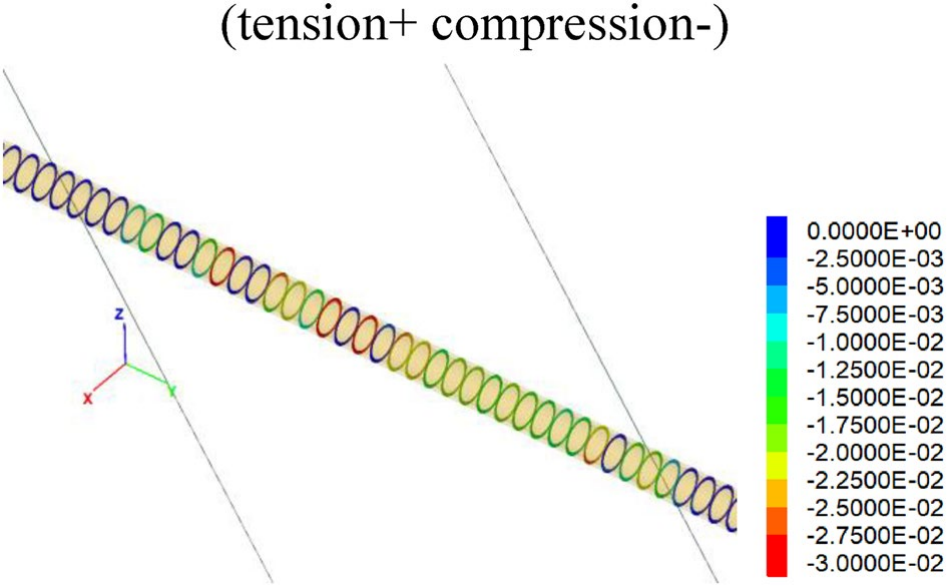
Normal relative deformation of hinged joints under 60 cm fortified dislocation.

**Figure 12:**
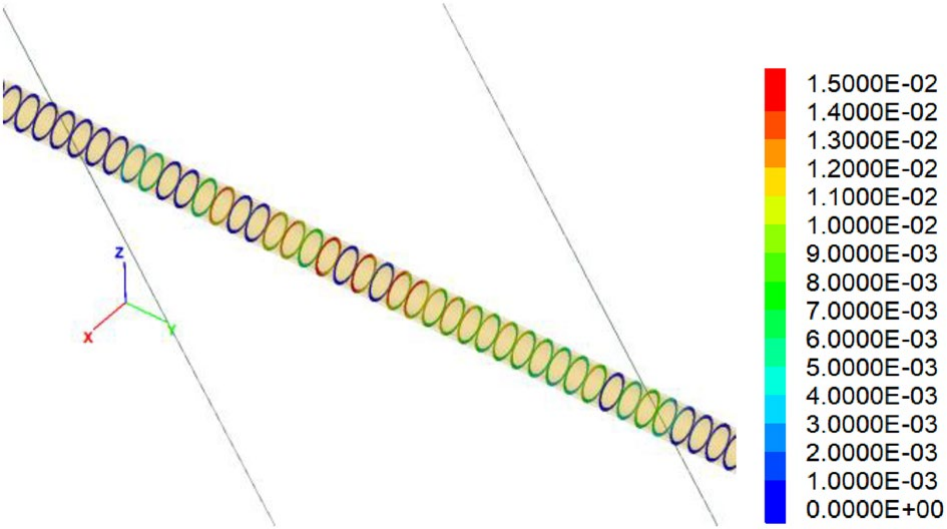
Tangential relative deformation of hinged joints under 60 cm fortified dislocation.

Figures 11 and 12 show the relative normal and tangential deformations of the hinged joints. Results show that the maximum normal deformation of the hinged joints is about 3.5 cm, which is located within the central segments of the fault zone. Similarly, the maximum tangential deformation is about 1.5 cm, which also occurs within the central segments of the fault zone. Importantly, the relative deformation of the hinged joint is less than the 5 cm reserved width.

### Tunnel displacement under different dislocation values

**Figure 13:**
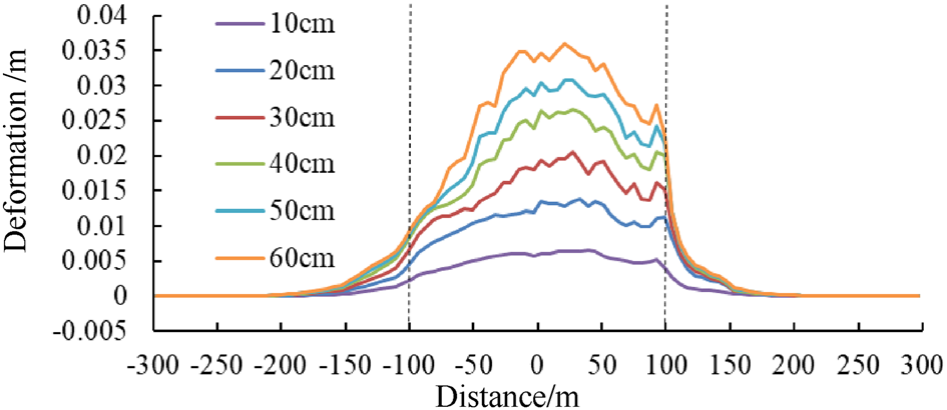
Curve of relative deformation of the left and right side walls of the tunnel under different dislocation values.

**Figure 14:**
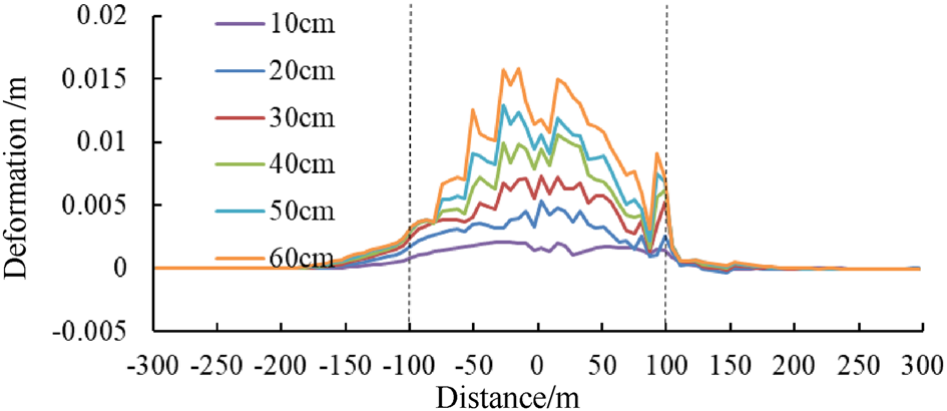
Curve of relative deformation of arched roof-arch bottom of the tunnel under different dislocation values.

Figures 13 and 14 shows the relative deformation between the left and right walls and between the vault and arch bottom of the tunnel under different dislocation values. These results show that relative deformation values basically escalate with an increase in fault dislocation, with the convergence of the left and right walls playing a dominant role, complemented by settlement of the vault towards the arch bottom. The maximum relative deformation of the tunnel is about 3.5 cm with the 60 cm dislocation value, which occurs within the central segment of the dislocation zone.

### Stress and internal force of tunnel under different dislocation values

**Figure 15:**
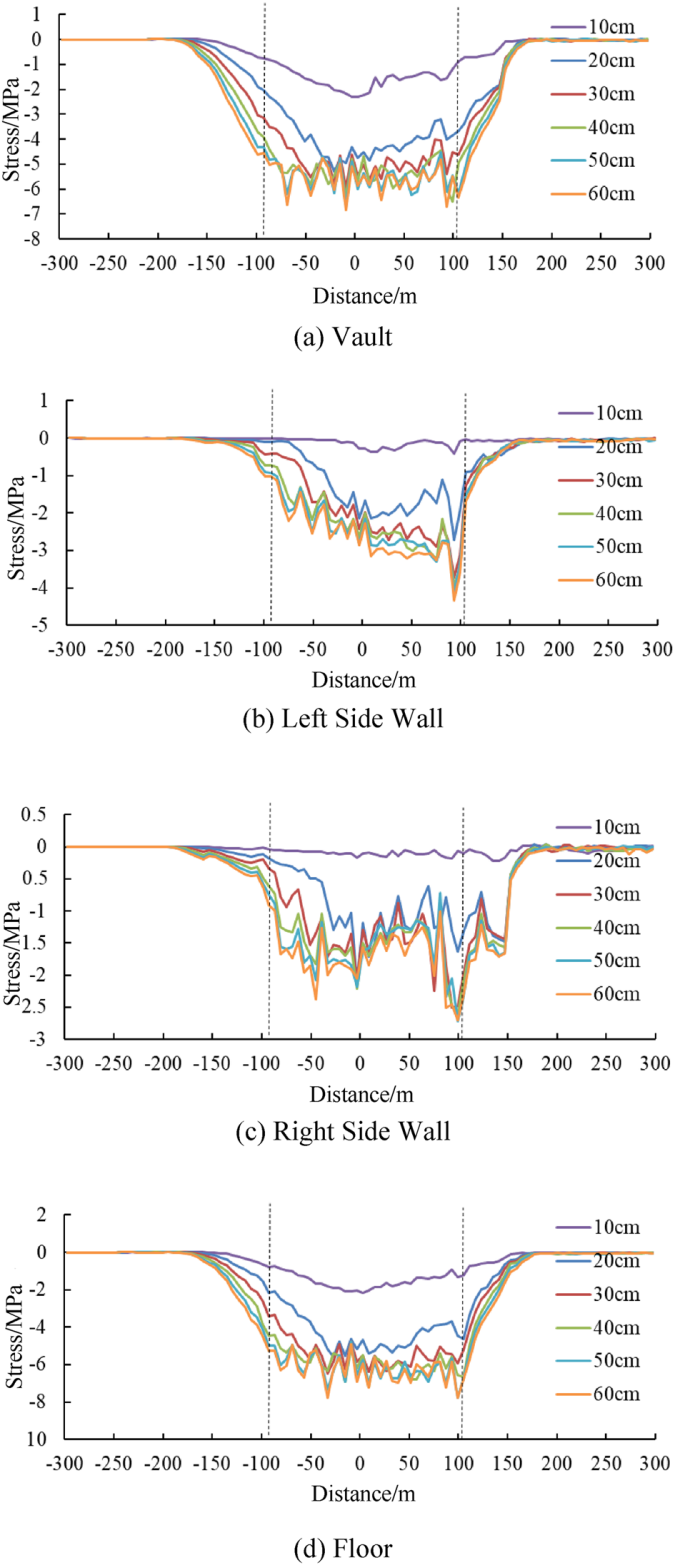
Change curve of the longitudinal stresses of articulated tunnel lining along the axial direction.

**Figure 16:**
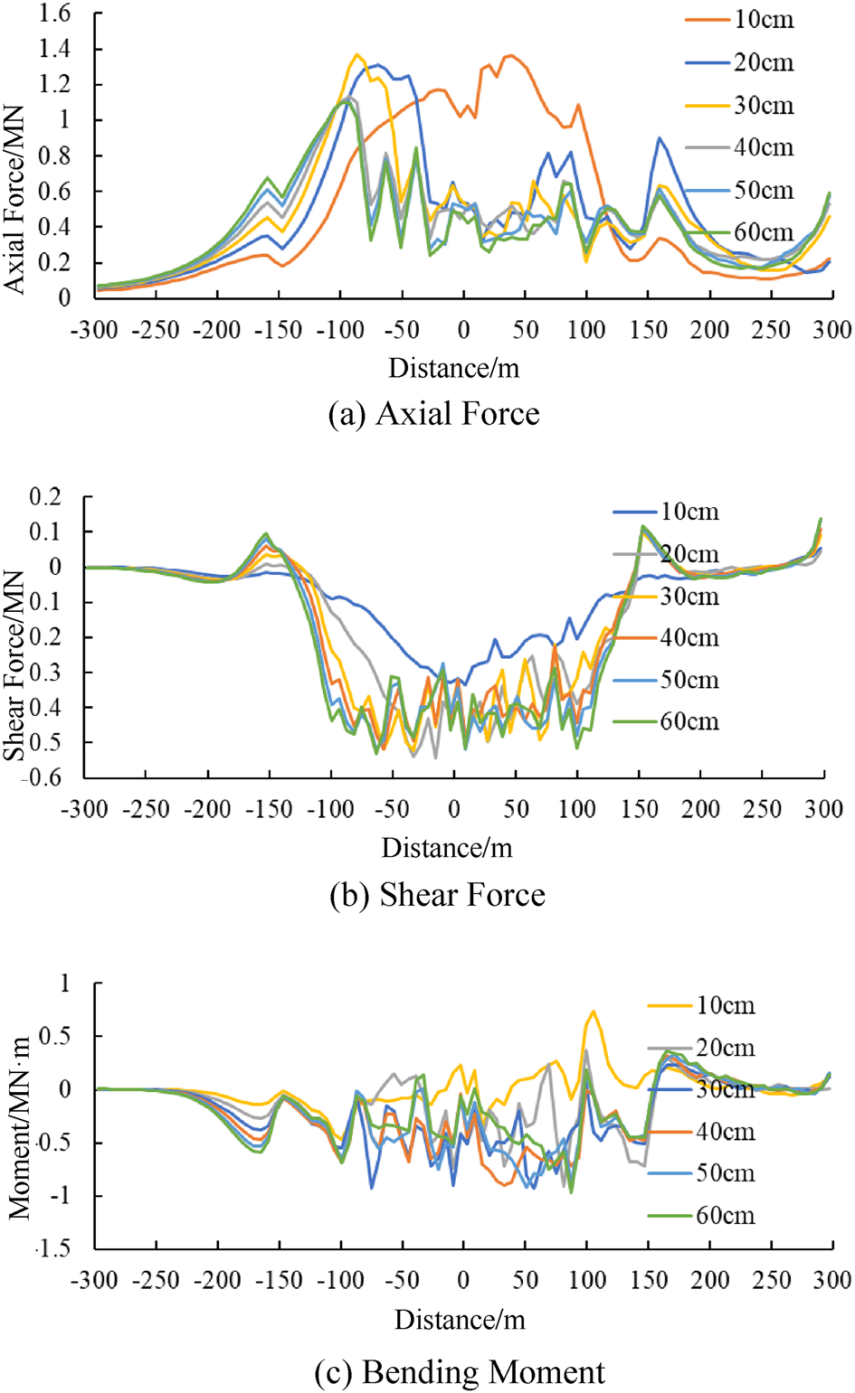
Change curve of internal forces of articulated tunnel lining along the axial direction.

Figure 15 shows the longitudinal stress in different parts of the tunnel under different dislocation values. While Fig. 16 shows the distribution of internal force values along the axial direction of tunnel, including axial force, horizontal bending moment and horizontal shear force, when the tunnel lining is treated as a flexible beam. Significant longitudinal stress and internal force values can be noted in the tunnel lining within the fault. As for the longitudinal stress, the longitudinal stress at the vault and arch bottom are larger that of the left and right side walls. the maximum longitudinal stress is about 5 ~ 6 MPa in those portions.

Both in Figs. 15 and 16, a similar phenomenon can be found that the stresses and internal forces does not tend to increase significantly with the dislocation after the dislocation reaches 30 cm. The possible reason for this is because the main deformation occurs in the hinged joint rather than the lining segment, after the 30 cm dislocation. This phenomenon is good for the stability of the tunnel, that the hinge joints are functioning, effectively improving the bearing state of the lining under fault dislocation.

## Discussion

Summarizing the above analysis results, the mechanism of the anti-dislocation measures can be obtained as follows:

The anti-dislocation measures effectively reduce the tensile force on the entire lining inside the fault zone, and has a certain degree of improvement on the compressive state at the junction with the fault zone. Under the condition of anti-dislocation measures, the bending moment caused by the misalignment of the lining also has a significant reduction trend. The anti-dislocation measures has a limited effect on reducing the shear force of the tunnel lining, and only in some sections, the shear force of the lining has a certain degree of reduction.

## Conclusions

In this paper, the anti-dislocation design of the Xianglushan Tunnel of the Central Yunnan Water Diversion Project is checked. Taking the Longpan-Qiaohou Fault F10-1 as a typical case.

1. The influence of the active fault of F10-1 on the anti-dislocation adaptive structure of the Xianglushan Tunnel is evaluated, and its effect on reducing the internal force and deformation of the lining is verified based on numerical calculation. The research results can be directly applied to the engineering design and construction of water conveyance tunnels crossing active faults, and provide favorable support for the construction of related tunnels.
2. The F10-1 Longpan-Qiaohou Fault is used as a typical representative to carry out the studies on surrounding rock, structural stability and anti-dislocation adaptability. The anti-dislocation measures considered are as follows: the segment length is 6 m; the hinged joint width is 5 cm, the fortified length is 1.5D, i.e., for a fault zone with a width of 200 m, it extends to the hanging wall and foot wall by 50 cm each, and the hinged joint is filled with plastic concrete materials with an elastic modulus of 500 MPa.
3. The results show that one side wall of the tunnel is under tension due to the fault movements mainly being strike-slip, and the tensile stress value is small, about 5 MPa. Results show that the maximum normal deformation of the hinged joints is about 3.5 cm, which is located between the central segments of the fault zone; and the maximum tangential deformation is about 1.5 cm, which also occurs between the central segments of the fault zone. The relative deformation of the hinged joints is less than the reserved width, i.e., 5 cm. The hinged design of adaptive structure can effectively improve the stress state of the lining under dislocation.

## Data Availability

The datasets used and/or analyzed during the current study available from the corresponding author on reasonable request.
